# Team dynamics in emergency surgery teams: results from a first international survey

**DOI:** 10.1186/s13017-021-00389-6

**Published:** 2021-09-16

**Authors:** Lorenzo Cobianchi, Francesca Dal Mas, Maurizio Massaro, Paola Fugazzola, Federico Coccolini, Yoram Kluger, Ari Leppäniemi, Ernest E. Moore, Massimo Sartelli, Peter Angelos, Luca Ansaloni, the Team Dynamics Study Group, Abubaker Abdelmalik, Nebyou Seyoum Abebe, Fikri M. Abu-Zidan, Yousif Abdallah Yousif Adam, Harissou Adamou, Antonino Agrusa, Emrah Akin, Henrique Alexandrino, Syed Muhammad Ali, Pedro Miguel Almeida, Francesco Amico, Michele Ammendola, Jacopo Andreuccetti, Daniel Aparicio-Sánchez, Antonella Ardito, Giulio Argenio, Ingolf Harald Askevold, Boyko Tchavdarov Atanasov, Goran Augustin, Selmy Sabry Awad, Carlo Bagnoli, Lovenish Bains, Dimitrios Balalis, Edoardo Baldini, Oussama Baraket, Mirko Barone, Jorge Arturo Barreras, Giovanni Bellanova, Helena Biancuzzi, Mark Brian Bignell, Roberto Bini, Daniele Bissacco, Paoll Boati, Andrea Bottari, Konstantinos Bouliaris, Antonio Brillantino, Luis Antonio Buonomo, Salvatore Buscemi, Valentin Calu, Riccardo Campo Dall’Orto, Joao Miguel Carvas, Gianmaria Casoni Pattacini, Fausto Catena, Valerio Celentano, Marco Ceresoli, Mircea Chirica, Pasquale Cianci, Nicola Cillara, Stefania Cimbanassi, Stefano Piero Bernardo Cioffi, Elif Colak, Luigi Conti, Silvia Dantas Costa, Fabrizio D’acapito, Dimitrios Damaskos, Koray Das, Richard Justin Davies, Andrew Charles De Beaux, Belinda De Simone, Zaza Demetrashvili, Andreas Kyriacou Demetriades, Stefano Denicolai, Giuseppe Di Buono, Isidoro Di Carlo, Salomone Di Saverio, Bogdan Diaconescu, Rigers Dibra, Sandra Dios-Barbeito, Agron Dogjani, Maurizio Domanin, Mario D’Oria, Virginia Duran Munoz-Cruzado, Barbora East, Gerald Takem Ekwen, Adel Hamed Elbaih, Juan Pablo Escalera-Antezana, Giuseppe Esposito, Roser Farre, Antonjacopo Ferrario di Tor Vajana, Vinicius Cordeiro Fonseca, Francesco Forfori, Laura Fortuna, Evangelos Fradelos, Gustavo P. Fraga, Pietro Fransvea, Mahir Gachabayov, Alain Garcia Vazquez, Wagih Mommtaz Ghannam, Rossella Gioco, Giorgio Giraudo, Mario Giuffrida, Michela Giulii Capponi, Carlos Augusto Gomes, Ricardo Alessandro Teixeira Gonsaga, Emre Gonullu, Jacques Goosen, Tatjana Goranovic, Ewen Alexander Griffiths, Muad Gamil Haidar, Hytham K. S. Hamid, Timothy Craig Harddastle, Matthias Hecker, Edgar Fernando Hernandez Garcia, Eduardo Cancio Huaman, Martin Hutan, Orestis Ioannidis, Arda Isik, Azzain Mahadi Hamid Ismail, Nizar Ismail, Ji Young Jang, Sujala Niatarika Rajsain Kalipershad, Lewis J. Kaplan, Yasin Kara, Evika Karamagioli, Aleksandar Karamarkovia, Alfie J. Kavalakat, Aristotelis Kechagias, Jakub Kenig, Jim S. Khan, Vladimir Khokha, Roberto Felix Klappenbach, Roberto Klappenbach, Yoshiro Kobe, Victor Kong, Dimitrios Korkolis, Hayato Kurihara, Akira Kuriyama, Aitor Landaluce-Olavarria, Leo Licari, Andrey Litvin, Varut Lohsiriwat, Claudia Cristina Lopes Moreira, Eftychios Lostoridis, Agustín Tovar Luna, Davide Luppi, Gustavo Miguel Machain, Marc Maegele, Daniele Maggiore, Ronald V. Maier, Mallikarjuna Manangi, Andrea Manetti, Baris Mantoglu, Federico Mariani, Athanasios Marinis, Evandro Antonio Sbalcheiro Mariot, Gennaro Martines, Aleix Martinez Perez, Pietro Mascagni, Damien Massalou, Renato Bessa Melo, Luca Miceli, Andrea Mingoli, Tushar S. Mishra, Ali Yasen Y. Mohamedahmed, Rajashekar Mohan, Dieter Morales-Garcia, Sami Mohamed Siddig Mustafa, Mukhammad David Naimzada, Ionut Negoi, Melkamu Kibret Nidaw, Giuseppe Nigri, Habeeb Damilola Ogundipe, Cristina Oliveri, Stefano Olmi, Leonardo Pagani, Giuseppe Palomba, Desire Pantalone, Arpad Panyko, Ciro Paolillo, Davide Papis, Nikolaos Pararas, Francesco Pata, Giovanna Pavone, Francesca Pecchini, Gianluca Pellino, Maria Pelloni, Andrea Peloso, Eduardo Perea Del Pozo, Rita Goncalves Pereira, Bruno Monteiro Pereira, Aintzane Lizarazu Perez, Gennaro Perrone, Antonio Pesce, Giovanni Petracca, Micaela Piccoli, Edoardo Picetti, Emmanouil Pikoulis, Tadeja Pintar, Giovanni Pirozzolo, Mauro Podda, Pietro Previtali, Francesca Privitera, Clelia Punzo, Martha Alexa Quiodettis, Niels Qvist, Razrim Rahim, Alexander Reinisch-Liese, Maria Rita Rodriguez-Luna, Daniel Roizblatt, Francesco Pietro Maria Roscio, Stefano Rossi, Boris Evgeniev Sakakushev, Juan Carlos Salamea, Ibrahima Sall, Fabrizio Sammartano, Alejandro Sanchez Arteaga, Sergio Sanchez-Cordero, Diego Sasia, Robert G. Sawyer, Charalampos Seretis, Mario Serradilla-Martin, Vishal G. Shelat, Sergei Shlyapnikov, Romeo Lages Simoes, Boonying Siribumrungwong, Mihail Slavchev, Leonardo Solaini, Gabriele Soldini, Kjetil Soreide, Larysa Sydorchuk, Ruslan Sydorchuk, Ali Muhammad Syed, Luis Tallon-Aguilar, Jih Huei Tan, Antonio Tarasconi, Dario Tartaglia, Nicola Tartaglia, John Taylor, Giovanni Domenico Tebala, Ricardo Alessandro Teixeira Gonsaga, Michel Teuben, Matti Tolonen, Giovanni Tomasicchio, Tania Triantafyllou, Giuseppe Trigiante, Victor Turrado-Rodriguez, Roberta Tutino, Matteo Uccelli, Bakarne Ugarte-Sierra, Mika Ukkonen, Panteleimon G. Vassiliu, Juan Manuel Verde, Massimiliano Veroux, Ramon Vilallonga, Diego Visconti, Maciej Waledziak, Tongporn Wannatoop, Lukas Werner Widmer, Michael Samuel James Wilson, Ting Hway Wong, Sofia Xenaki, Byungchul Yu, Andee Dzulkarnaen Zakaria, Diego A. Zambrano, Monica Zese

**Affiliations:** 1 Department of Clinical, Diagnostic and Pediatric Sciences, University of Pavia, Polo Didattico “Cesare Brusotti” Viale Brambilla, 74, 27100 Pavia, Italy; 2 IRCCS Policlinico San Matteo Foundation, General Surgery, Viale Camillo Golgi, 19, 27100 Pavia, Italy; 3 Department of Management, Lincoln International Business School, University of Lincoln, Lincoln, UK; 4 Department of Management, Ca’ Foscari University, Venice, Italy; 5 Department of Surgery, University of Pisa, Pisa, Italy; 6 General, Emergency and Trauma Surgery, Pisa University Hospital, Pisa, Italy; 7 Department of General Surgery, Rambam Health Care Campus, Haifa, Israel; 8 Abdominal Center, University Hospital Meilahti, Helsinki, Finland; 9 Shock Trauma Center at Denver Health, Denver, CO USA; 10 Department of General Surgery, Macerata’s Hospital, Macerata, Italy; 11 Department of Surgery and MacLean Center for Clinical Medical Ethics, The University of Chicago, Chicago, IL USA

**Keywords:** Trauma teams, Knowledge translation, Team dynamics, Non-technical skills, Trauma leaders

## Abstract

**Background:**

Emergency surgery represents a unique context. Trauma teams are often multidisciplinary and need to operate under extreme stress and time constraints, sometimes with no awareness of the trauma’s causes or the patient’s personal and clinical information. In this perspective, the dynamics of how trauma teams function is fundamental to ensuring the best performance and outcomes.

**Methods:**

An online survey was conducted among the World Society of Emergency Surgery members in early 2021. 402 fully filled questionnaires on the topics of knowledge translation dynamics and tools, non-technical skills, and difficulties in teamwork were collected. Data were analyzed using the software R, and reported following the Checklist for Reporting Results of Internet E-Surveys (CHERRIES).

**Results:**

Findings highlight how several surgeons are still unsure about the meaning and potential of knowledge translation and its mechanisms. Tools like training, clinical guidelines, and non-technical skills are recognized and used in clinical practice. Others, like patients’ and stakeholders’ engagement, are hardly implemented, despite their increasing importance in the modern healthcare scenario. Several difficulties in working as a team are described, including the lack of time, communication, training, trust, and ego.

**Discussion:**

Scientific societies should take the lead in offering training and support about the abovementioned topics. Dedicated educational initiatives, practical cases and experiences, workshops and symposia may allow mitigating the difficulties highlighted by the survey’s participants, boosting the performance of emergency teams. Additional investigation of the survey results and its characteristics may lead to more further specific suggestions and potential solutions.

## Introduction

Hospital trauma teams are made up of a wide range of healthcare practitioners who collaborate to provide high-quality care. While many scholars agree that a good trauma team’s qualities are self-evident, there is little quantitative evidence on the most desirable attributes associated with good trauma care [1, 2]. Moreover, team dynamics in trauma and emergency settings are crucial. Trauma teams are multidisciplinary and need to work under great pressure and with time constraints, often with little knowledge about the trauma’s causes and the patient’s identity, pre-existing conditions, and wishes. The emergency setting does not often allow the investigation of existing clinical literature and guidelines or consultation with other colleagues for second opinions.

Trauma teams so need to employ processes and tools to allow effective knowledge translation and sharing among their members and, eventually, with the patient whenever possible. Knowledge translation can be described as the ability to translate concepts between various contexts by stakeholders with different skills, objectives, and even feelings [3]. In healthcare and surgery, knowledge translation looks particularly challenging, as different practitioners (e.g., physicians, nurses, technicians, researchers) and specialists need methodologies, organizational processes, and resources to effectively communicate and exchange information among themselves [4] and with patients [5].

During trauma situations, trauma team preparation has highlighted the importance of non-technical skills like teamwork and leadership. Non-technical cognitive skills are related to critical task success. Increased focus on non-technical skills during trauma team training proved to contribute to better long-term success in trauma scenarios. Both team leaders and team members need decision-making and situational awareness skills, which should be discussed explicitly to boost results [6].

Another crucial skill for trauma leaders and team members has been identified as communication, which has proved to be a necessary component to ensure and facilitate safe teamwork and prevent errors and mistakes, especially when teams are multidisciplinary and in emergency settings [7]. Miscommunication among trauma team members is two to four times more likely to result in fatal errors than among other medical teams [8, 9]. Despite the recognized importance of communication in trauma team training, a Swedish study found that developing a secure and effective verbal communication mode in interdisciplinary teams remained challenging, especially when it comes to decision-making [7].

Nevertheless, the role between the trauma team and the patient has been evolving over time. Shared decision-making [10] and patient engagement [11–13], which originated in non-surgical disciplines, have become widely accepted methods of decision-making in various medical fields and are particularly useful when there are more valid treatment options to be taken into consideration. The literature highlights how patients’ satisfaction increases once they are engaged in choosing their clinical options [14]. Given the growing importance of patient autonomy in modern healthcare and the role that physicians play in supporting that autonomy, it is ethically important to make decisions that take the patient’s values and priorities into account [10]. Apart from some extremely acute decisional problems, many decisions in trauma surgery are preference-sensitive, so no particular procedure is obviously preferable for all patients with that condition. As a consequence, certain options look particularly suitable for shared decision-making. A national survey conducted among Dutch trauma surgeons highlighted the importance of shared decision making and patient engagement to foster good patient care and patients’ satisfaction. Still, surgeons showed a strong misunderstanding of what these concepts entail, and they report difficulties in their implementations [10].

Starting from these premises, this paper aims to deepen the team dynamics in emergency surgery, by conducting an international survey promoted by the World Society of Emergency Surgery. Topics like knowledge translation dynamics and tools, the relevance of non-technical skills, and the general difficulties for trauma teams to work together to improve patient care are investigated through an online questionnaire.

## Methods

### Design and setting

This exploratory study of international trauma surgeons used a population-based online survey to gather demographic, knowledge and practice-based information regarding their team dynamics.

In January, an e-mail invitation to participate in the survey was sent out from the World Society of Emergency Surgery and shared on the society’s website and Twitter profile. Three reminders followed through the same channels.

The survey was conducted in English through Google Forms, and followed the Checklist for Reporting Results of Internet E-Surveys (CHERRIES) [15].

The electronic questionnaire was created starting from a research protocol shared within the steering committee. Most questions were linked to previous studies in the fields of trauma and emergency surgery [10, 16, 17], knowledge management and organization science [3, 18, 19], and clinical ethics [4, 17, 20, 21].

Before the invitations were sent out, the electronic questionnaire was reviewed and filled in by a sample of surgeons.

The invitation e-mail included detailed information about the survey’s subject and goals, its expected duration (< 15 min), and the possibility to join the Team Dynamics Study Group to continue investigating and sharing the results. All the responses were anonymous, as well as who the investigators had been.

Soon after the closure of the investigation, the final dataset was downloaded into an excel spreadsheet file. No Institutional Review Board (IRB) approval was sought.

### Survey

The first questions aimed at describing the sample, including the gender, the number of years of experience in trauma surgery, the kind of institution (academic vs non-academic), the country, the position held, the eventual inclusion within a trauma team (institutionalized or not, and of which kind), the type of trauma leader, the courses attended, and the presence of diverse team members. Most of such questions were gathered from Woltz et al. [10], and Reichert et al. [16].

The questions about knowledge translation aimed at testing the surgeons’ awareness about such a concept, first with an open question and then investigating the translation tools and facilitators. Surgeons were asked to give their opinion about the effectiveness of some tools [3] with a 5-point Likert scale and identifying the ones used in their institutions.

The questions about non-technical skills asked the participants to rank the importance of 10 skills gathered from Massaro et al. [19] using a 5-point Likert scale, and then with an open question about the importance of non-technical skills in facilitating the work within Trauma Teams.

One more question was an open one about the main difficulties for Trauma Teams to work together.

### Statistical analysis

Quantitative data were analyzed using summary statistics; qualitative (free-text) data were categorized, and frequencies of categories were reported [10]. The statistical analysis was conducted using the software R [22, 23].

## Results

### Participants

A total of 402 trauma surgeons participated in the survey, filling in all the required answers. The following Table 1 reports some descriptive statistics about the sample.

**Table 1:** Descriptive statistics about surgeons and institutions participating in the study

	Number	Percent
Participants	402	100.00
*Gender*		
Male	338	84.08
Female	61	15.17
Prefer not to answer	3	0.75
*Kind of institution*		
Academic	292	72.64
Non academic	110	27.36
*Current position*		
Head of department	60	14.93
Senior consultant	171	42.54
Board-certified surgeon	109	27.11
Resident	62	15.42
*Part of a trauma team*		
Yes	320	79.60
No	82	20.40
*Role of the trauma leader*		
A surgeon	211	52.49
An anesthesiologist/intensivist	77	19.15
An emergency physician	90	22.39
Others	24	5.97
*Part of a diverse team*		
Yes	250	62.19
No	152	37.81

**Fig. 1:**
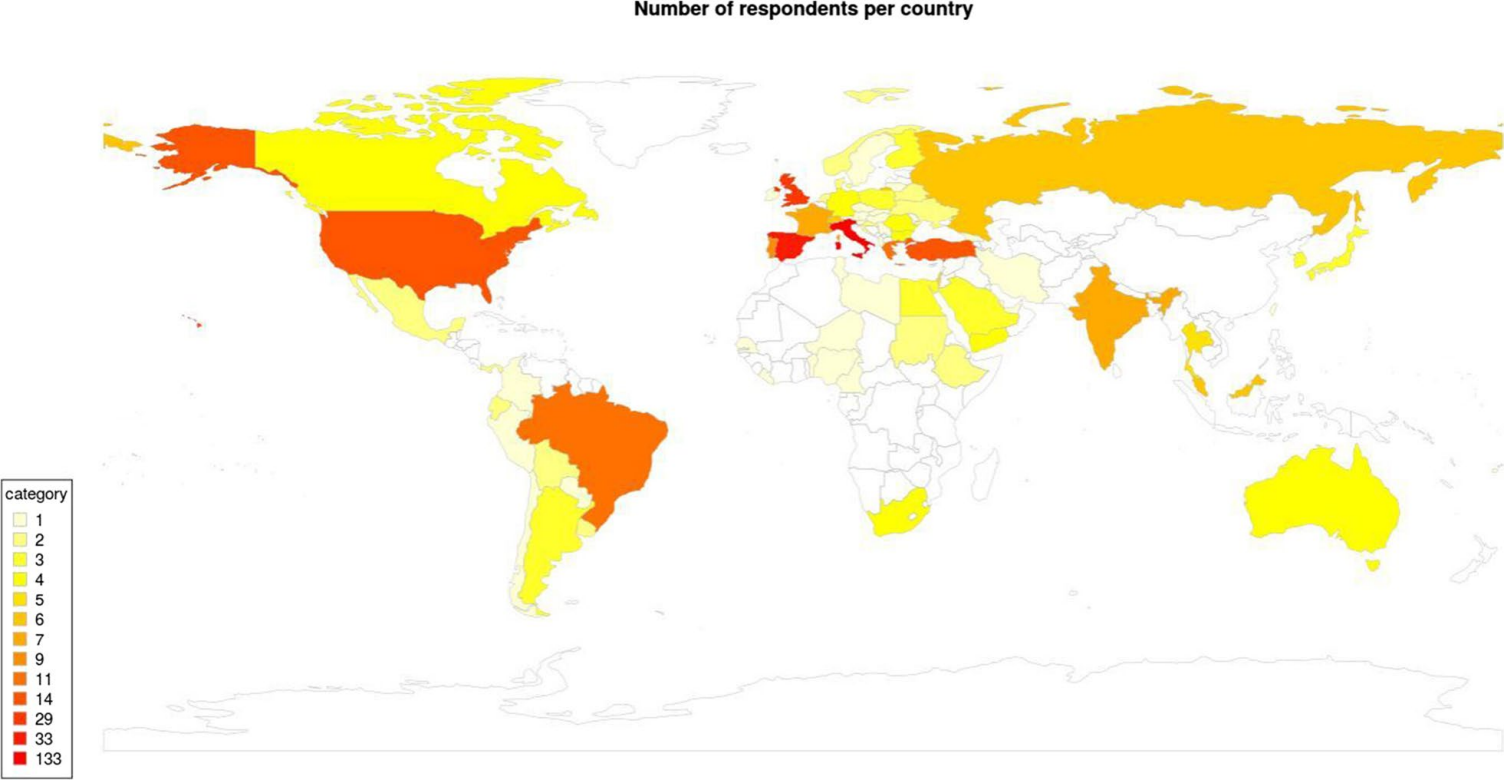
Respondents per country

Figure 1 illustrates the countries involved in the study. Results show how most of the respondents come from Europe (66% of respondents). North and South America account respectively for 5% and 6%. Asian countries see the majority of respondents concentrated in Russia and India. In all, the respondents are well distributed around the world, even though results from African trauma teams were underrepresented.

**Fig. 2:**
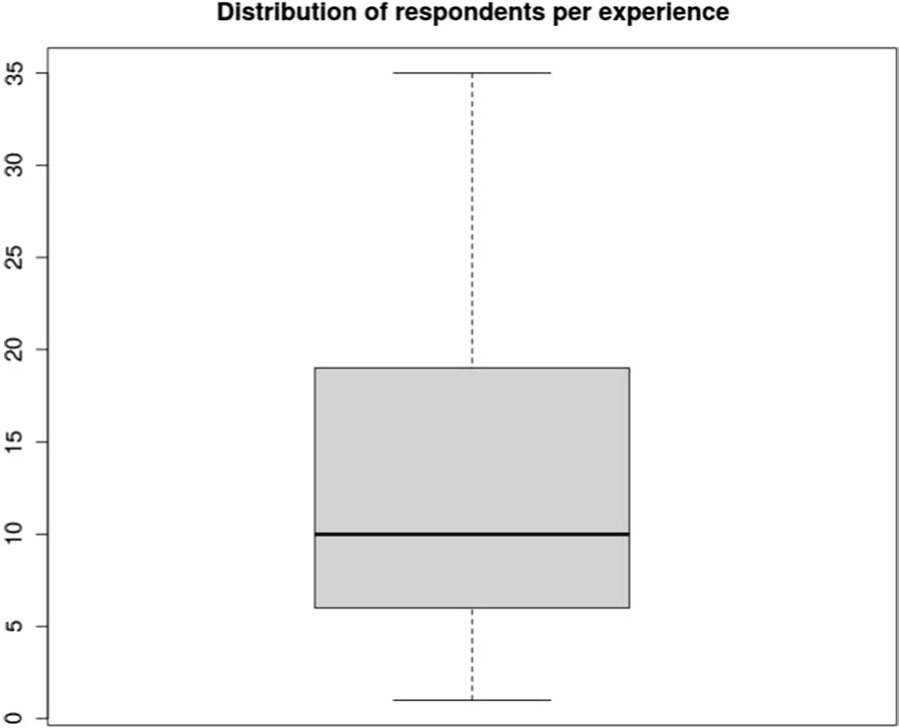
Respondents per years of experience

Figure 2 describes the years as a trauma surgeon, highlighting a median of 10 years of experience, ranging from 1 to 35 years of emergency surgery and the majority of respondents (from the first to the third quartile) ranging from 6 to 18 years of experience.

### Definition of knowledge translation

The surgeons gave various definitions of knowledge translation. Two researchers (LC and FDM) rated each statement as concordant, discordant, or inconclusive, following the analysis of Woltz et al. [10] and the definition of knowledge translation gathered from Dal Mas et al. [3]. More than half of the participants (223, 55% of the total sample) gave responses rated as concordant, stressing the need to translate knowledge into practice and to pass knowledge and information among colleagues, also in a multidisciplinary perspective. 87 (22% of the total respondents) definitions were considered discordant, as they recalled different concepts or could only show a partial view of the phenomenon. The remaining 92 participants (23% of the sample) were rated as inconclusive, because it was impossible to determine their concordance: the answers were too short to be able to interpret their exact meaning, or they only contained a synonym of the term knowledge translation. Of those, many surgeons declared that they had never heard the term before, or they were unsure about its meaning. Table 2 shows examples of given answers that were rated as concordant, discordant and inconclusive [10].

**Table 2:** Examples and way of rating of given answers to the question: what is your understanding of knowledge translation?

Rated as	Given answer	Reason for rating
Concordant	“The ability to translate theoretical knowledge into real-life scenarios”	The descriptions recall the idea of transforming and transferring knowledge into something different in another context: from theory to clinical practice, from academia and laboratories to organizations and people, among team members
	“Process of moving research knowledge into clinical practice”	
	“The transfer of knowledge from academia and laboratories into organizations and people who can make use of it”	
	“Knowledge translation is the process of implementation of theoretical and clinical knowledge and skills in clinical practice and their impact on patient outcomes”	
	“Medicine should be based on knowledge translation, which is the process of moving research from the laboratory into the hands of doctors who can put it to practical use. This is particularly important in surgery as it links theoretical knowledge and research to practice”	
	“It is the ability to make knowledge accessible to different stakeholders by translating it into various contexts”	
	“Translating concepts in a different context to transfer and share knowledge”	
Inconclusive	“Working together”	Too short
	“Everything”	Not linked to the concept of knowledge translation
	“Very important”	
	“Sharing knowledge”	Only a synonym
	“I do not know what it is”	Unknown
Discordant	“Ethical approach to work”	Ethics can be linked to knowledge translation but does not describe the concept
	“Training, through continuing and continuing education”	Training can be defined as a knowledge translation tool but does not describe the concept
	“It means applying daily guidelines in clinical practice”	Clinical guidelines can be defined as knowledge translation tools but do not describe the concept
	“Treat the patient as yourself”	The connection with the transformation, sharing and transfer of knowledge is missing
	“Understanding responsibility”	
	“It is a combination of surgical skills, information, leadership skills and personal example”	
	“The ability to use diagnostic and therapeutic protocols during an emergent event.”	
	“Quality of service”	
	“The ability to know how to manage every situation”	

**Fig. 3:**
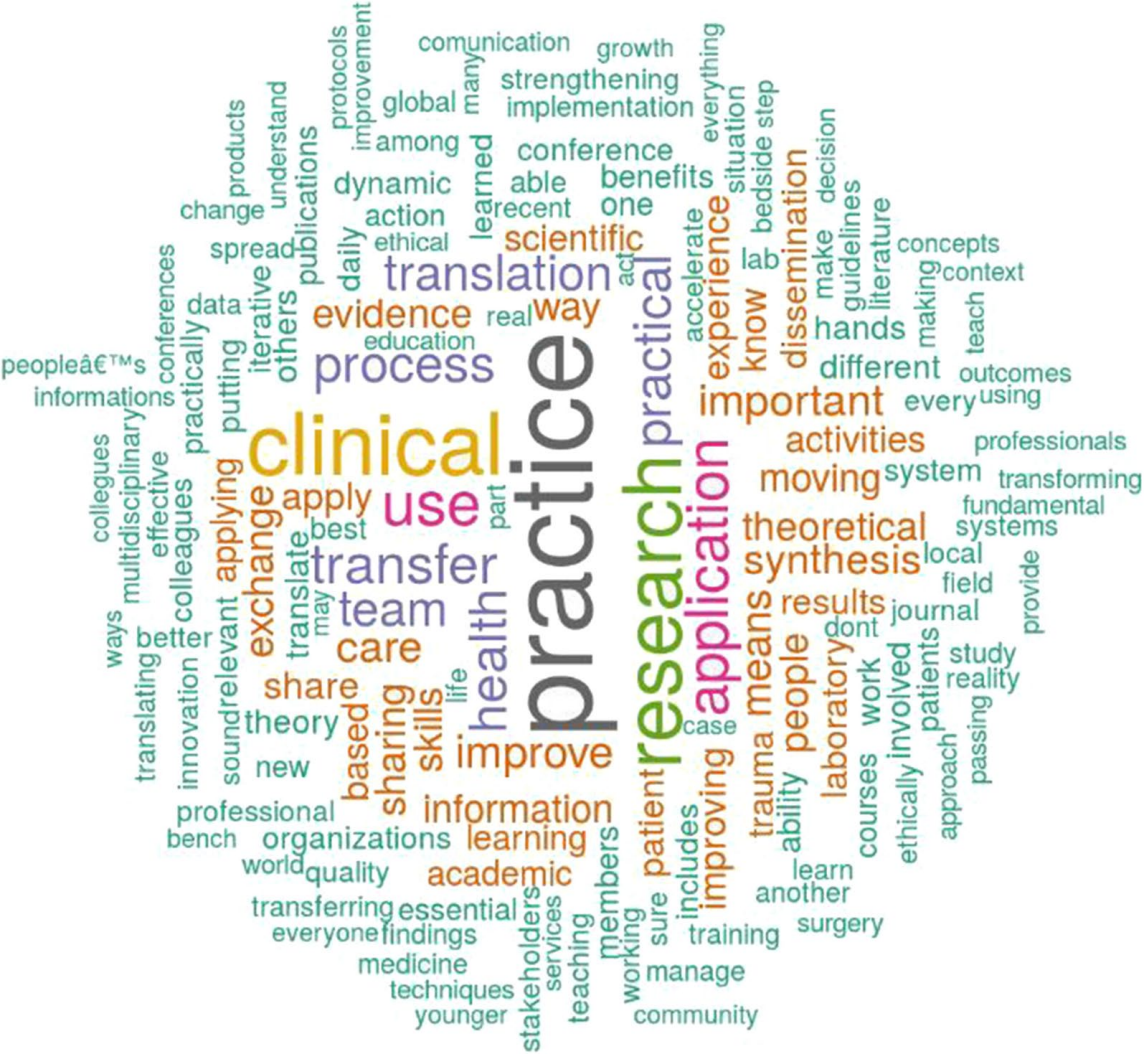
Word cloud analysis: the concept of knowledge translation

Figure 3 shows the word cloud gathered from extracting the respondents’ keywords to describe the concept of knowledge translation. To create the word cloud, first, we translated the definitions not provided in English (e.g. from Spanish, Italian, and Russian to English). Then, we cleaned the text from stopwords and numbers (e.g. commas, question marks, etc.). Second, we translated all the sentences in lower letter and converted derived words using a common stemming English procedure (e.g. “speak,” “speaks,” “speaking” were grouped together). All the process was developed using the software R and the packages “wordcloud” and “tm” [23]. The word cloud was created with the top 158 concepts named by at least three respondents, excluding “knowledge.” Results show that the most used words recall the ideas of transfer and practical application of health and clinical processes. Interesting concepts mentioned in the description are activities, synthesis, exchange, dissemination, scientific, sharing, and skills.

**Fig. 4:**
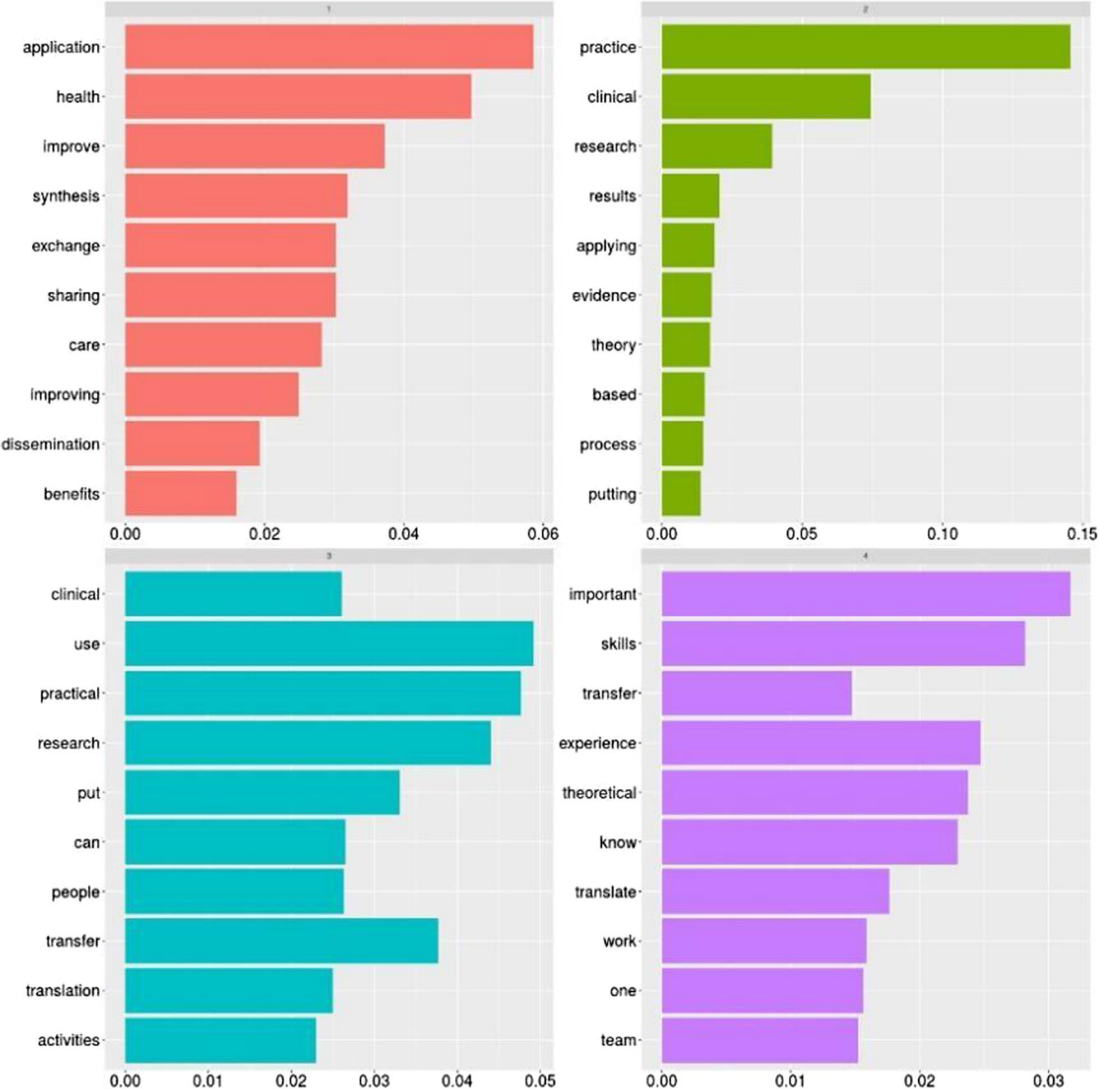
Topic modelling analysis: the concept of knowledge translation

From the pure word cloud, we then developed a topic modelling analysis to summarise the main concepts related to the definition of knowledge translation given by the participants. Using computer-aided content analysis, we analyzed the data’s content using R and its topic modelling feature to identify the key topics retrieved from the given description. Content analysis [24] is a versatile methodological framework that aids in textual data organization by allowing categorizing and coding. Topic modelling [25] stands as a statistical method used in the content analysis to find abstract “topics” that appear in a set of documents. To identify and code text into unique subjects, the Latent Dirichlet Allocation (LDA) method was used [26]. The first identified topic (labelled in red) recalls the application, synthesis, and exchange of knowledge to improve the processes and care. Another topic (marked in green) reminds the need to transfer and share information within the team. One more topic (light blue) recalls the translation of research into practice by people (being them researchers/scholars or clinicians). Similarly, the purple topic stresses the application, improvement, and transfer of theory into clinical practice (Fig. 4).

### Knowledge translation tools

**Fig. 5:**
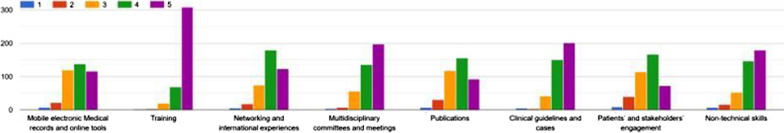
Knowledge translation tools per importance

The participants were requested to rate the perceived importance of some knowledge translation enablers, as defined by Dal Mas et al. [3], using a 5-point Likert scale where 1 = not relevant at all and 5 = very relevant. Results are summarised in the following Fig. 5.

Results highlight the high importance recognized to training, multidisciplinary committees and meeting, clinical guidelines and cases, and non-technical skills. Interesting enough, less importance is paid to patients and stakeholders’ engagement and publications.

Participants were then asked to select the tools that they used in their practice, regardless of their personal opinion on those. Results are reported in the following Table 3. The findings highlight that most surgeons (respectively, 85% and 77%) use clinical guidelines and cases and training in their daily practice. Around half of them employs electronic records and online tools, multidisciplinary groups, publications, and non-technical skills. Interesting enough, only 23% of them declare to engage with patients and other stakeholders actively.

**Table 3:** Knowledge translation tools used

Tools	Number	Percent
Mobile electronic medical records and online tools	203	50.50
Training	309	76.87
Multidisciplinary committees and meetings	199	49.50
Networking and international experiences	242	60.20
Publications	235	58.46
Clinical guidelines and cases	343	85.32
Patients’ and stakeholders’ engagement	91	22.64
Non-technical skills	227	56.47

### Non-technical skills

The role of non-technical skills [6] is assessed by asking the participants about the effective relevance of such skills, with 373 surgeons (93%) confirming their importance in emergency surgery. An open question asked the surgeons to argue about the reason for their answer.

**Fig. 6:**
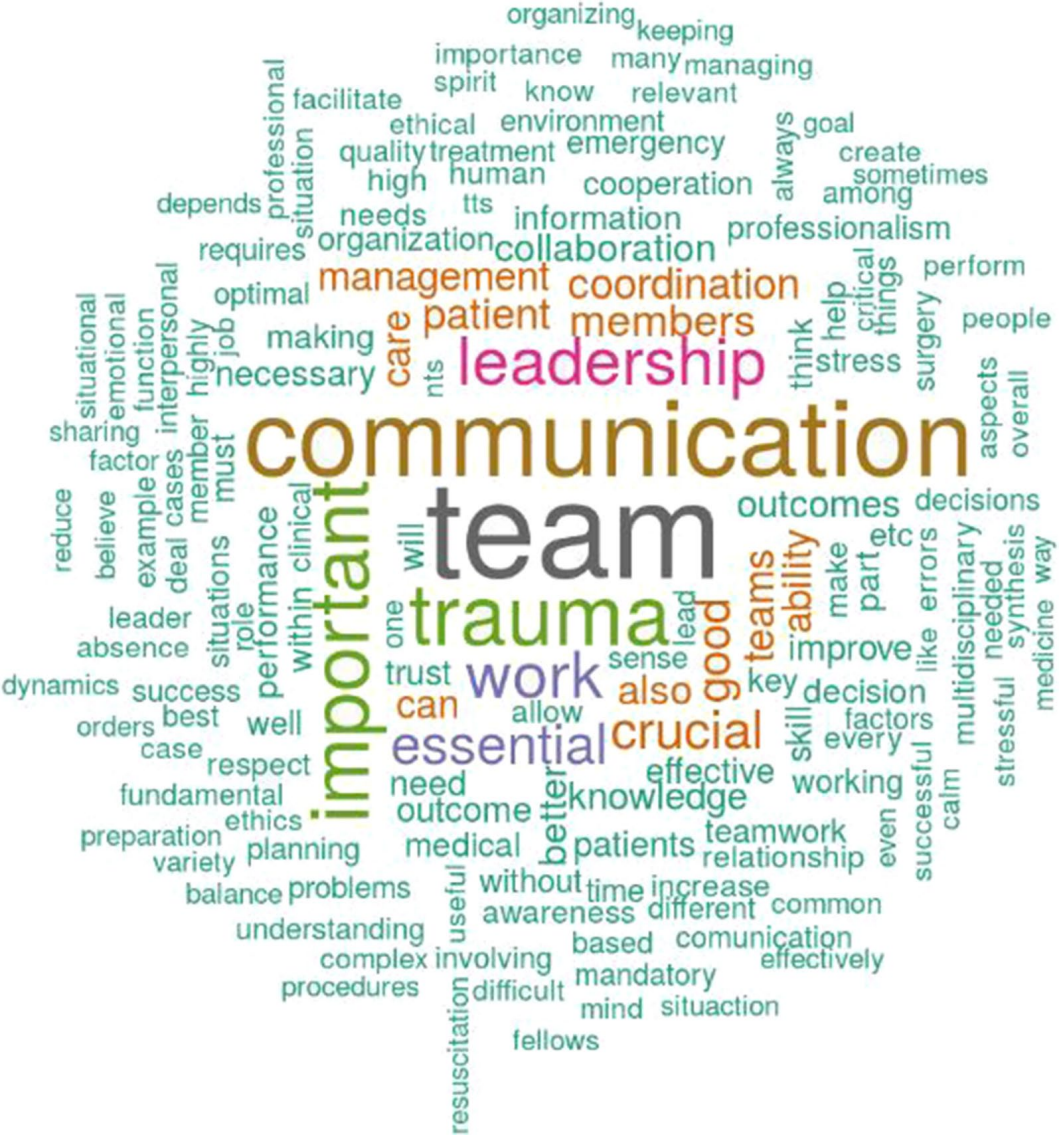
Word cloud analysis: the relevance of non-technical skills

Figure 6 reports the word cloud gathered from the analysis, with the top 158 concepts named by at least three participants, excluding “non,” “technical,” “non-technical,” and “skills.” The most used words recall the ideas of communication and leadership within the trauma team. Interesting concepts mentioned in the description are management, coordination, team members, care, and patients. Adjectives like “crucial,” “essential,” and “good” are used to stress the concept.

**Fig. 7:**
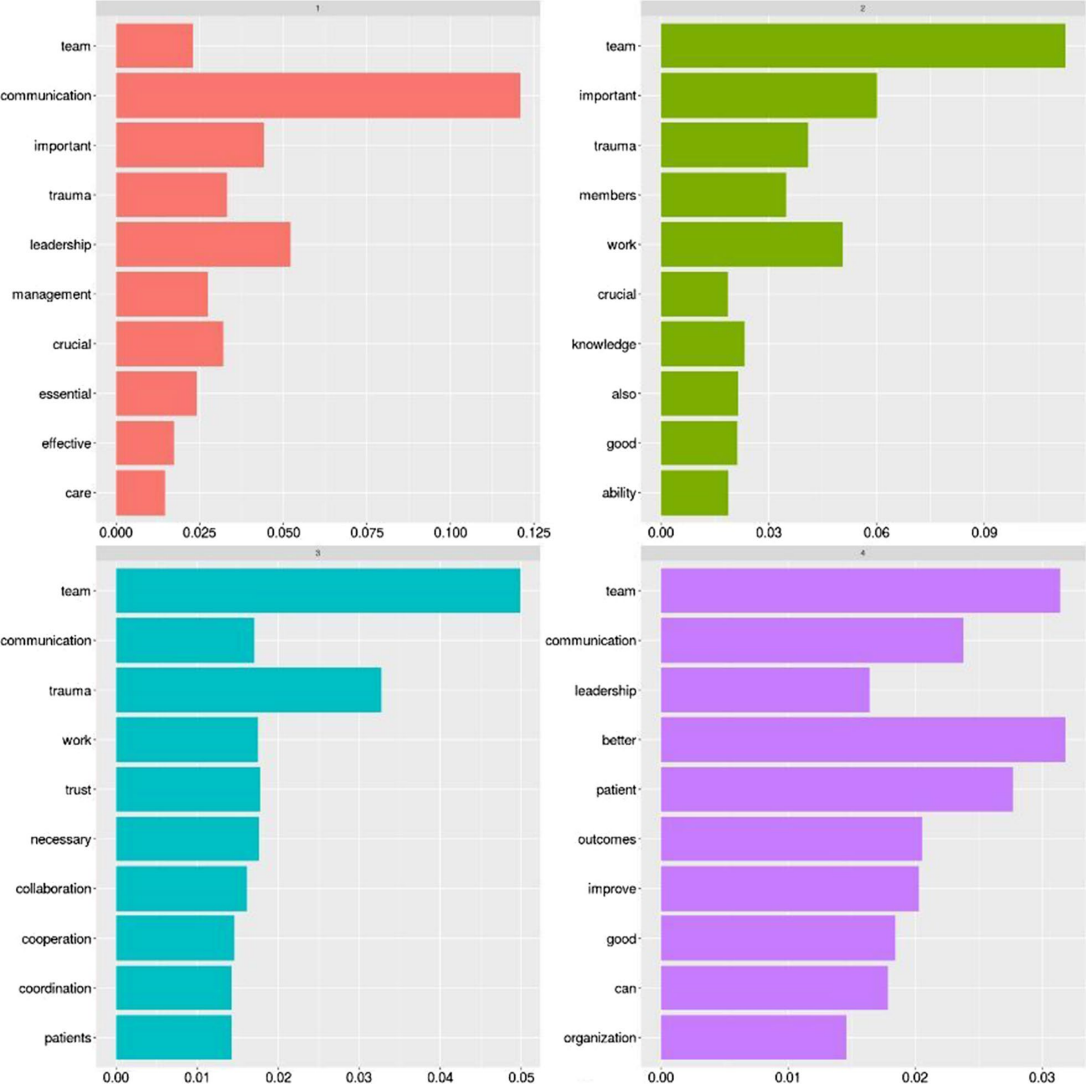
Topic modelling analysis: the relevance of non-technical skills

Similar results can be gathered from the topic modelling analysis, as reported in the following Fig. 7. Again, communication and leadership play a central role as the main non-technical skills to facilitate emergency surgery teams’ work.

### Main difficulties for trauma teams

One last question asked the participants to freely describe the main difficulties encountered by trauma teams to gather effectively.

**Fig. 8:**
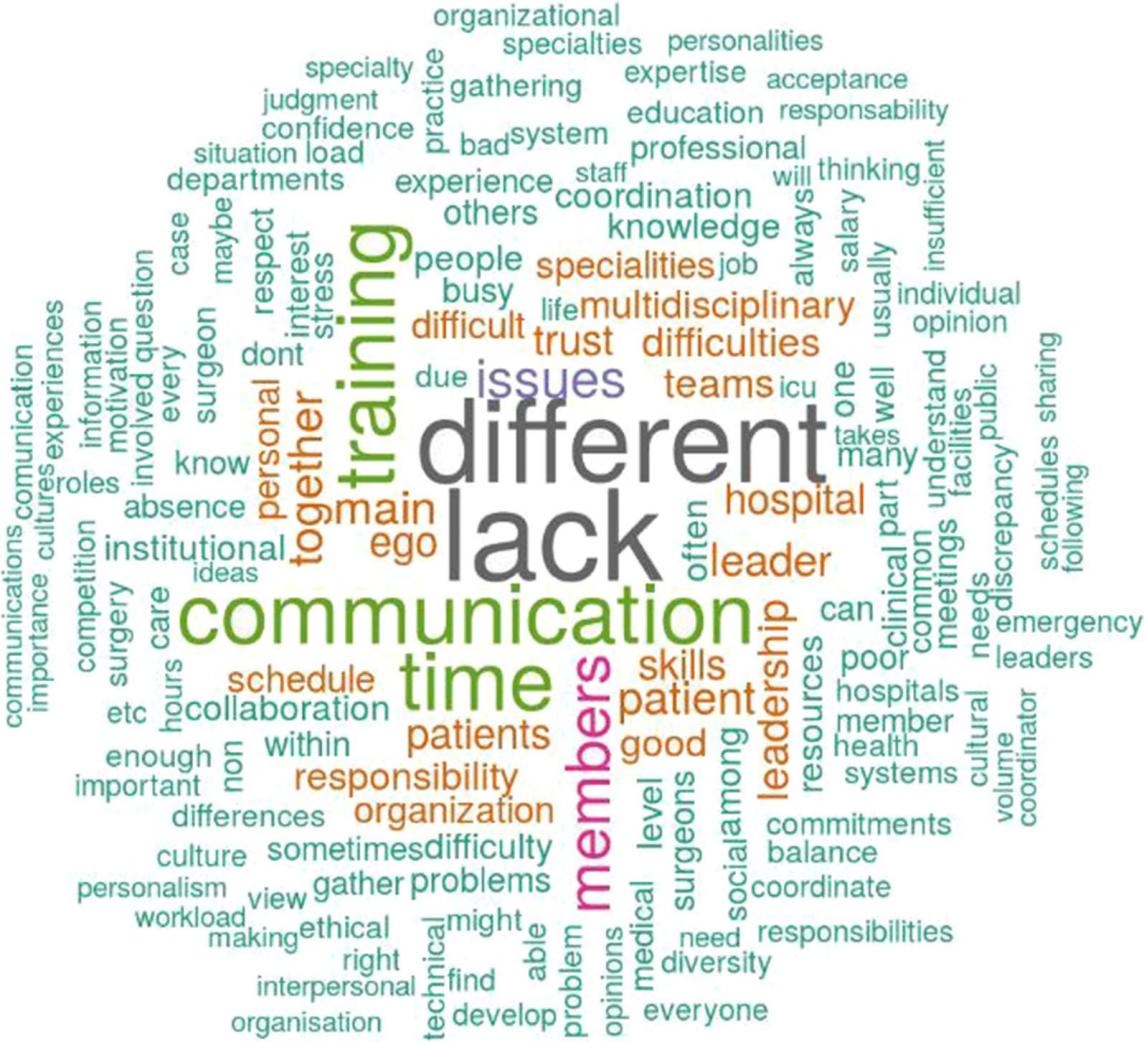
Word cloud analysis: the main difficulties for trauma teams

Figure 8 shows the word cloud gathered from the analysis, with the top 158 concepts named by at least three participants, excluding “trauma,” “team(s),” “work,” and “working.” The most used words recall a lack of time, communication, and training. Interesting concepts mentioned in the description are difficulties, trust, leaders, ego, responsibility, schedule, stress, and multidisciplinarity. Some participants also mentioned the challenges related to the management of the COVID-19 pandemic and its related disruptions.

**Fig. 9:**
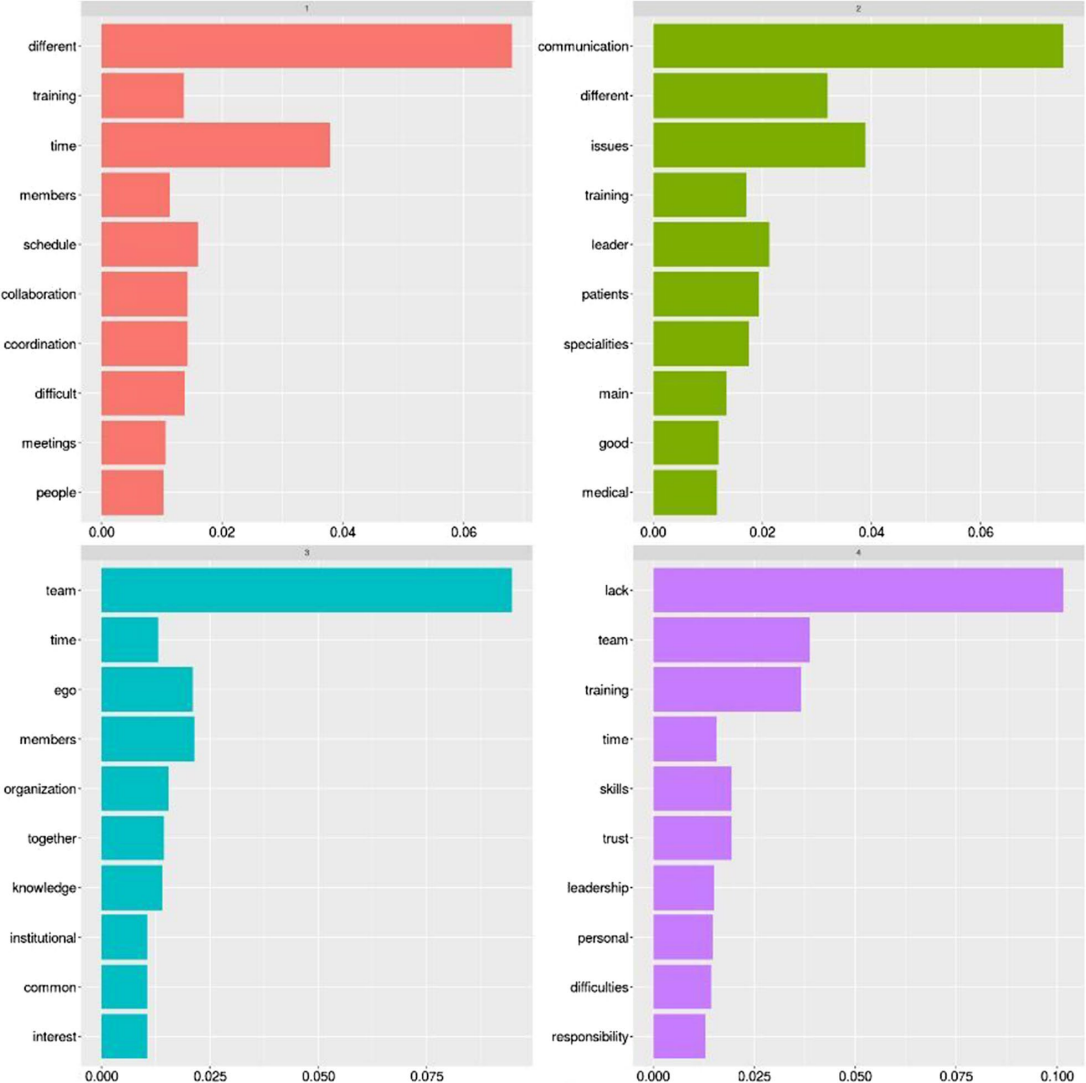
Topic modelling analysis: the main difficulties for trauma teams

Similar concepts can be gathered from the topic modelling analysis, as reported in the following Fig. 9.

## Discussion

Trauma and emergency surgery can be defined as a challenging setting for a variety of reasons: time constraints, lack of information about the traumatic event and the patient conditions, and the need to put more specialities and skills at work. Trauma teams should then employ knowledge translation mechanisms and tools to transfer and share information effectively, often relying on non-technical skills like leadership, teamwork, and communication. Clinical decision making appears crucial, and it may also involve the patient whenever possible.

The results of our international survey among trauma surgeons offer exciting insights on the abovementioned issues.

Regarding knowledge translation dynamics, most emergency surgeons are aware of the concept and its meaning. Still, most of them stress more the “bench to bedside” effect rather than its validity in all clinical processes, especially when multidisciplinary staff members are involved [3, 4]. Moreover, half of the surgeons do not have a clear idea about the meaning of translation, as they report not knowing much about the topic, or they have only a partial view of it. Still, moving continuously back and forth among theory, training, and clinical practice appeared fundamental in their understanding. The lack of consensus about the awareness and meaning of knowledge translation calls for dedicated training and dissemination activities, like workshops, dedicated congress tracks, scientific journals’ calls for papers, and symposia. Sharing practical cases may also stand as an effective strategy to disseminate the potential of knowledge translation mechanisms to boost the team’s performance and outcomes [4].

Responses about the knowledge translation tools, as recognized by the literature [3, 4], reveals several appealing results. Almost all of the survey’s participants recognize the value of training, which stands as one of the World Society of Emergency Surgery’s core values. Non-technical skills, clinical guidelines, and multidisciplinary teams are also mentioned as relevant. Interestingly enough, a lack of consensus emerges when it comes to patients’ and stakeholders’ engagement, as surgeons seem to have opposite personal views. If these results may depend on a variety of circumstances (age, type of education, kind of institution, geographical location, among others), the type of tools effectively used in the clinical practice follow a similar trend. Indeed, clinical guidelines and training are largely employed by the sample. Only around half of the surgeons reported using electronic records and online tools, multidisciplinary committees and meetings, publications, networking with international colleagues, and non-technical skills (especially communication) in their daily practice. Such results seem in contrast with the latest trends in surgery, which highlight, for instance, the increasing use of new technologies, e-health, telemedicine and online tools [27–29], including Artificial Intelligence and Machine Learning [30, 31] in supporting surgical decision making [32], and the recognized importance of non-technical skills in trauma surgery [6, 9].

Confirming the analysis of Woltz et al. [10], less than one out of four surgeons reported engaging with the patients and other stakeholders in daily practice. While this may depend on the type of emergency or disaster to manage, shared decision making and patients’ engagement appear to be more difficult to implement. Our results suggest that a need for surgeon education and training in shared decision making and patients’ engagement skills emerge. Increasing awareness about the ethical importance of the topic may foster joint decision-making in trauma surgery, contributing to patients’ satisfaction [10–12].

Surgeons recognize several difficulties in conducting their work within teams. Problems are highlighted in the trust among colleagues, in authoritarian relations with leaders, in the presence of a strong ego, heavy responsibilities, schedules, and stress, also connected with the COVID-19 emergency [33–37]. Interestingly enough, in contrast with what the literature claims [3, 4, 38], multidisciplinarity is mentioned more as a liability than as an asset. Scientific societies should address such difficulties in offering dedicated training and support, both during residency and continuous clinical education throughout a surgeon’s career. Again, knowledge translation mechanisms and facilitators [3] and a “one size does not fit all” approach [4] may mitigate several of such difficulties.

## Conclusions

Team dynamics appear fundamental in trauma and emergency surgery. Our international survey underlined how multidisciplinary trauma teams need to operate under great pressure, and tailored mechanisms and processes should be put in place to facilitate teamwork and performance. Dynamics involve both the work within teams, as well as the relationship with the patients. While several difficulties emerge, especially during pandemic times, mitigation actions are required by scientific societies through training and practical tools and solutions.

Further analysis on our data may offer practical insights dividing the sample according to unique participants’ characteristics (such as years of experience as an emergency surgeon, role within the trauma team, being part of a diverse group or an academic versus non-academic institution, gender), to see if results change or are confirmed, and therefore if different solutions can be offered to different professionals. Although the sample may be appropriate, it is not equally distributed from a geographical perspective, since most of the participants come from developed countries like those in Europe, America, and Australia. Further investigation would be needed in low-income or developing countries.

Moreover, the particular time in which the survey was conducted, in a “new normal” following the COVID-19 pandemic among surgical disruptions and the stimulus towards new technological solutions, can offer unique opportunities to implement modern tools and practices.

## Data Availability

The datasets used and/or analyzed during the current study are available from the corresponding author upon reasonable request.
