# Correction to: Team dynamics in emergency surgery teams: results from a first international survey

**DOI:** 10.1186/s13017-021-00397-6

**Published:** 2021-11-01

**Authors:** Lorenzo Cobianchi, Francesca Dal Mas, Maurizio Massaro, Paola Fugazzola, Federico Coccolini, Yoram Kluger, Ari Leppäniemi, Ernest E. Moore, Massimo Sartelli, Peter Angelos, Fausto Catena, Luca Ansaloni, the Team Dynamics Study Group

**Affiliations:** 1 Department of Clinical, Diagnostic and Pediatric Sciences, University of Pavia, Polo Didattico “Cesare Brusotti” Viale Brambilla, 74, 27100 Pavia, Italy; 2 General Surgery, IRCCS Policlinico San Matteo Foundation, Viale Camillo Golgi, 19, 27100 Pavia, Italy; 3 Department of Management, Lincoln International Business School, University of Lincoln, Lincoln, UK; 4 Department of Management, Ca’ Foscari University, Venice, Italy; 5 Department of Surgery, University of Pisa, Pisa, Italy; 6 General, Emergency and Trauma Surgery, Pisa University Hospital, Pisa, Italy; 7 Department of General Surgery, Rambam Health Care Campus, Haifa, Israel; 8 Abdominal Center, University Hospital Meilahti, Helsinki, Finland; 9 Shock Trauma Center at Denver Health, Denver, CO USA; 10 Department of General Surgery, Macerata’s Hospital, Macerata, Italy; 11 Department of Surgery and MacLean Center for Clinical Medical Ethics, The University of Chicago, Chicago, IL USA; 12 General and Emergency Surgery, Bufalini Hospital, Cesena, Italy

## Correction to: World J Emerg Surg (2021) 16:47 10.1186/s13017-021-00389-6

The original article [1] has been corrected to restore Fausto Catena to the main authorship after having been mistakenly removed during production.
